# Supplementary material for: Radiotherapy continuity for cancer treatment: Lessons learned from natural disasters
Source: PLoS One. 2025 Sep 3;20(9):e0308056. doi: 10.1371/journal.pone.0308056 (PMC12407550; doi:10.1371/journal.pone.0308056)
Supplement: S1 Text — (PDF) [file pone.0308056.s001.pdf]

Supporting information 1

Literature summary

In the following, the literature identified in the SLR is presented in alphabetical order by author.

Anacak et al. document the impact of consecutive earthquakes of magnitudes 7.8, 7.5, and 6.4 in Turkey and northern Syria, resulting in healthcare infrastructure destruction, staff casualties, and disruptions in 15 radiotherapy departments. Despite minimal damage to radiotherapy bunkers and hardware, shielded bunker doors were dislocated, and power failures led to the loss of medical device calibrations. Collaboration with neighbouring centres enabled the resumption of treatments within days, with full operational recovery achieved in two weeks. Volunteer support supplemented exhausted staff, and assessments ensured the integrity of buildings, electronic systems, LINAC calibrations, dosimetry systems, and lasers before radiotherapy treatment reinitiation [1].

Espinel et al. detail experiences from four Atlantic hurricanes, focusing on the US, each presenting distinct hazards, including storms, extreme rainfall, tornadoes, and floods. Even with experience from past events, power grid collapses caused hospital outages, patient evacuations, and disruptions to critical supplies. The hurricanes also delayed cancer care due to staff and infrastructure challenges, resulting in disrupted screenings, delayed detection, and poorer prognoses. Concurrently, the COVID-19 pandemic compounded challenges for radiotherapy centres [2].

Gay et al. draw from their experience during Hurricane Maria in Puerto Rico, which brought destructive wind, torrential rain, and landslides. The hurricane led to power outages, disrupted transportation, contamination of drinking water, and limited access to medical care and treatments. The loss of communication infrastructure hampered coordination and access to information. The hurricane's impact on healthcare facilities

was significant, with radio oncology clinics facing treatment disruptions and delayed operations [3].

Grew et al. describe their encounter with superstorm Sandy in the US, emphasising clinical decision-making, patient safety, and radiotherapy service restoration. The flooding of a radiotherapy department caused extensive equipment damage, including several LINACs, necessitating new radiotherapy planning due to destroyed immobilisation devices [4].

Joob and Wiwanitkit document a major flooding in Asia impacting 12 million individuals over two months, leading to inoperable cancer care units in 20 hospitals and flooding within radiotherapy centres. The disaster resulted in transportation challenges for patients, medical supplies, and drugs. Treatment delays and restricted access to radiotherapy centres compounded the crisis [5].

Lopez-Araujo et al. report on Hurricane Maria in Puerto Rico revealed extensive devastation, including the destruction of high-voltage networks, resulting in a complete power outage. The hurricane's aftermath severely impacted radiotherapy centres, with 11 out of 18 centres rendered inaccessible after a week, causing a reduction in patient numbers to 20-30% of normal operation in the remaining functioning centres [6].

Man et al. reviewed the literature until November 2016, examining the impact of natural disasters on cancer care. They found that disaster events substantially disrupt oncology services, causing damage to infrastructure, communication systems, and medication supply. Challenges include workforce management, medical records, tissue sample loss, patient and staff evacuation, communication disruption, and medication shortages. The authors highlighted the prevalence of medical record losses due to water damage or collapsed infrastructure and identified psychological stress as a concern for patients. The study underscores the need for natural disaster preparedness in oncology care and acknowledges the limited evidence on the impact of disasters on cancer patient survival rates [7].

Mireles et al. detail their experience with three US tropical storms, destroying treatment machines, a CT, a brachytherapy device, quality assurance equipment, and

patient records. The hospital lost power, including emergency generators, due to water damage. Evacuations, regional damage, and prolonged power outages disrupted operations. They describe the implementation of disaster response plans, including a command centre establishment and readiness assessment [8].

Ozaki and Tsubokura highlight challenges in accessing cancer care, particularly radiation therapy, following an earthquake-triggered tsunami. While most radiation oncology centres resumed services within a month, swift recovery was attributed to equipment maintenance companies and collaborative efforts among neighboring hospitals and physicians. Patients faced prolonged travel for treatment, reflecting persistent demographic changes and disaster-related healthcare disruptions [9].

Pérez-Andújar recounts remote experience during Hurricane Maria in Puerto Rico, highlighting days of uncertainty, power grid failure, disrupted water systems, and complete communication breakdown. Puerto Ricans turned to social media for information provision. Shared desperation, anxiety, and helplessness prevailed, compounded by struggles for resources. They cite treatment challenges and patients' delayed access to hospitals due to geographical constraints. The author emotionally emphasises the vulnerability of radiotherapy cancer treatment [10].

Roach et al. state that every effort should be made to restart thoracic radiation treatments for lung cancer patients affected by a disaster as soon as feasible [11].

Royce et al. emphasise the significant threat posed to radiotherapy departments by hurricanes and associated flooding, citing examples of Hurricane Matthew in 2016 and Hurricane Florence in 2018, causing severe inundation in North Carolina. A regional radiation oncology centre, treating around 1300 patients annually, was affected and became inoperative during Hurricane Florence. Logistical challenges and transportation disruptions further compounded the impact [12].

References

1. Anacak Y, Kurtul N, Nasuhbeyoğlu D, Oymak E, Önal HC. Radiotherapy facilities after the Türkiye-Syria earthquakes: lessons from the tragedy. The Lancet

Oncology 2023; 24(4):312–4. Available from: URL: https://www.thelancet.com/journals/lanonc/article/piis1470-2045(23)00099-2/fulltext.

2. Espinel Z, Nogueira LM, Gay HA, Bryant JM, Hamilton W, Trapido EJ et al. Climate-driven Atlantic hurricanes create complex challenges for cancer care. The Lancet Oncology 2022; 23(12):1497–8. Available from: URL: https://www.thelancet.com/journals/lanonc/article/piis1470-2045(22)00635-0/fulltext.

3. Gay HA, Santiago R, Gil B, Remedios C, Montes PJ, López-Araujo J et al. Lessons Learned From Hurricane Maria in Puerto Rico: Practical Measures to Mitigate the Impact of a Catastrophic Natural Disaster on Radiation Oncology Patients. Practical Radiation Oncology 2019; 9(5):305–21. Available from: URL: https://www.sciencedirect.com/science/article/pii/s1879850019300797.

4. Grew D, Vatner R, DeWyngaert K, Nicholas S, Formenti S. The Impact of Superstorm Sandy on the Care of Radiation Oncology Patients. International Journal of Radiation Oncology Biology Physics 2013; 87(2):S490-S491.

5. Joob B, Wiwanitkit V. Lesson for management of cancerous patient in the big flooding. Journal of Cancer Research and Therapeutics 2012; 8(1):165–6. Available from: URL: https://journals.lww.com/cancerjournal/fulltext/2012/08010/lesson_for_management_of_cancerous_patient_in_the.45.aspx.

6. Lopez-Araujo J, Burnett OL. Letter from Puerto Rico: The State of Radiation Oncology After Maria's Landfall. International Journal of Radiation Oncology, Biology, Physics 2017; 99(5):1071–2.

7. Man RX-G, Lack DA, Wyatt CE, Murray V. The effect of natural disasters on cancer care: a systematic review. The Lancet Oncology 2018; 19(9):e482-e499.

8. Mireles M, Pino R, Teh BS, Farach A, Joseph A, Butler EB. Radiation Oncology in the Face of Natural Disaster: The Experience of Houston Methodist Hospital. International Journal of Radiation Oncology, Biology, Physics 2018; 100(4):843–4.

9. Ozaki A, Tsubokura M. Radiation Oncology and Related Oncology Fields in the Face of the 2011 ”Triple Disaster” in Fukushima, Japan. International Journal of Radiation Oncology, Biology, Physics 2018; 100(4):845–8.

10. Pérez-Andújar A. Puerto Rico: After María. International Journal of Radiation Oncology, Biology, Physics 2018; 100(4):834–5.

11. Roach MC, Robinson CG, Bradley JD. Natural Disasters and the Importance of Minimizing Subsequent Radiation Therapy Interruptions for Locally Advanced Lung Cancer. International Journal of Radiation Oncology, Biology, Physics 2018; 100(4):836–7.

12. Royce TJ, Papagikos MA, Maguire PD, Marks LB. Carolina Hurricanes. International Journal of Radiation Oncology, Biology, Physics 2019; 103(3):775–6.
