# Supplementary material for: Radiotherapy continuity for cancer treatment: Lessons learned from natural disasters
Source: PLoS One. 2025 Sep 3;20(9):e0308056. doi: 10.1371/journal.pone.0308056 (PMC12407550; doi:10.1371/journal.pone.0308056)
Supplement: S2 Text — (PDF) [file pone.0308056.s002.pdf]

Supporting information 2

Coding data

The following tables present the taxonomy, which consists of themes and code groups.

Each code group's description and the code frequency are also shown.

Table 1. Organisation theme, code groups and code frequency.

Theme	Group	Description	Codes
Organisation	Planning	Develop a robust emergency operations plan including proactive measures, preparing for cancer services even after natural disasters with long-lasting effects, and minimising the impact of therapy interruptions on patients.	4
Organisation	Preparation	Prepare for the sudden loss of key utilities at home and in the facility; apply proactive measures protecting against weather impacts and securing sufficient staff at duty.	5
Organisation	Control	Establish a command centre for centralised communication with patients, staff, coworkers, and authorities. Coordinate treatment alternatives.	3
Organisation	Leadership	Guide teams that are exposed to psychological and physical pressure with strong leadership and good morale; identify special support needs to provide early intervention.	3

Table 2. Collaboration theme, code groups and code frequency.

Theme	Group	Description	Codes
Collaboration	Network	Create a collaborative network of radiotherapy centres with regional task forces and healthcare coalitions.	7
Collaboration	Referral	Transfer patients to cooperating radiotherapy centres to continue treatment based on shared health records; consider offering housing and interpretation services.	7
Collaboration	Volunteers	Organise with the help of radiotherapy associations volunteer staff and aid workers to support or replace own staff; consider special care teams to support patients at home.	7
Collaboration	Partners	Engage with vendors, partners, and insurance companies to support emergency offers.	1

Table 3. Communication theme, code groups and code frequency.

Theme	Group	Description	Codes
Communication	Towards patients	Consider alternative communication methods, such as Social Media and information provision via the Internet or radio; also prepare for psychological support in patient communication.	4
Communication	Towards professionals	Secure backup communication for leadership and emergency plan execution; also prepare for psychological support for caregivers while maintaining an overall positive attitude.	4
Communication	Between professionals	Secure backup communication methods for communication between caregivers.	1

Table 4. Access, protection and therapy themes, code groups and code frequency.

Theme	Group	Description	Codes
Access	Transportation	Offer free transportation for patients to radiotherapy centres, utilising reserved fuel stocks.	2
Protection	Safeguard	Assure patient's safety and acute needs and safeguard inpatients to ensure uninterrupted access to care.	2
Protection	Evacuation	Protect patients and staff by executing prepared evacuation plans.	1
Therapy	Prolongation	Extend the centre's operation hours to evenings and weekends.	6
Therapy	Fractions	Consider compensating additional treatment fractions or using hypofractionation techniques to reduce the number of treatment fractions.	4
Therapy	Stop and resume	Pause scheduled radiotherapy treatments and conduct quality assurance of medical devices before resuming treatments.	2

Table 5. Facility and data themes, code groups and code frequency.

Theme	Group	Description	Codes
Facility	Provision	Secure and shut down sensitive equipment and materials in a controlled manner and protect against harm, particularly by potential flooding.	2
Facility	Building	House radiotherapy centres in one-floor buildings following strict building codes instead of placing them in multistorey hospital buildings.	1
Facility	Redundancy	Twin linear accelerators to minimize the need to recalculate treatment plans in case of device loss.	1
Facility	Electricity	Secure electricity supply with an emergency generator, not shared with others, with sufficient fuel storage, and protected against direct and indirect disaster impacts.	
Facility	Accommodation	Offer accommodation for those unable to commute or being evacuated.	2
Facility	Radiation	Evaluate alternatives for displaced bunker doors securing radiation protection.	1
Data	EHR	Use Electronic Health Records (EHR) with tested recoverable backups in an online repository and data links to co-operating radiotherapy centres.	8
Data	Patient	Provide the patient with updated records during their treatment course.	1
