# Supplementary material for: Radiotherapy continuity for cancer treatment: Lessons learned from natural disasters
Source: PLoS One. 2025 Sep 3;20(9):e0308056. doi: 10.1371/journal.pone.0308056 (PMC12407550; doi:10.1371/journal.pone.0308056)
Supplement: S3 Text — (PDF) [file pone.0308056.s003.pdf]

Supporting information 3

Survey data

The below tables present additional results of the online survey. The assessment of risk mitigation measures is shown for different themes and in descending order of T2B agreement values.

Table 1. Assessment of organisational risk mitigation (n=111, single selection).

**Risk mitigation measures**	**1**	**2**	**3**	**4**	**5**
Transferring patients to cooperating radiotherapy centres to continue treatment based on shared health records and considering offering housing and interpretation services is ...	62	34	10	3	2
Creating a collaborative network of radiotherapy centres with regional task forces and healthcare coalitions is ...	46	41	15	4	5
Engaging with vendors, partners, and insurance companies to support emergency offers is ...	42	39	20	7	3
Organise with the help of radiotherapy associations volunteer staff and aid workers to support or replace own staff and consider special care teams to support patients at home is ...	31	33	28	17	2

1: Very important, 2: Important, 3: Neutral, 4: Not important, 5: Not at all important

Table 2. Assessment of communication risk mitigation (n=111, single selection).

**Risk mitigation measures**	**1**	**2**	**3**	**4**	**5**
Securing Backup communication for leadership and emergency plan execution and preparing for psychological support for caregivers while maintaining an overall positive attitude is ...	44	51	10	2	4
Securing backup communication methods for communication between caregivers is ...	45	48	13	1	4
Considering alternative communication methods towards patients, such as Social Media and information provision via the Internet or radio, and preparing for psychological support in patient communication is ...	28	49	24	5	5

1: Very important, 2: Important, 3: Neutral, 4: Not important, 5: Not at all important

Table 3. Assessment of access, protection, therapy risk mitigation (n=111, single selection).

Risk mitigation measures	1	2	3	4	5
Protecting patients and staff by executing prepared evacuation plans is ...	70	32	3	2	4
Pausing scheduled radiotherapy treatments and conducting quality assurances of medical devices before resuming treatments is ...	57	42	8	2	2
Assuring patient's safety and acute needs and safeguarding inpatients to ensure uninterrupted access to care is ...	55	39	12	3	2
Compensating additional treatment fractions or using hypofractionation techniques to reduce the number of treatment fractions is ...	33	55	16	4	3
Offering free transportation for patients to radiotherapy centres, utilising reserved fuel stocks is ...	28	40	30	9	4
Extending the centre's operation hours to evenings and weekends is ...	9	38	41	14	9

1: Very important, 2: Important, 3: Neutral, 4: Not important, 5: Not at all important

Table 4. Assessment of facility and data risk mitigation (n=111, single selection).

Risk mitigation measures	1	2	3	4	5
Securing and shutting down sensitive equipment and materials in a controlled manner and protecting against harm, particularly by potential flooding, is ...	61	40	6	1	3
To twin linear accelerators to minimise the need to recalculate treatment plans in case of device loss is ...	56	42	9	1	3
Securing electricity supply with an emergency generator, not shared with others, with sufficient fuel storage, and protected against direct and indirect disaster impact is ...	47	33	21	4	6
Using Electronic Health Records (EHR) with tested recoverable backups in an online repository and data links to cooperating radiotherapy centres is ...	43	37	24	5	2
Offering accommodation for those unable to commute or being evacuated is ...	39	40	22	6	4
Providing the patient with updated records during their treatment course is ...	28	47	26	6	4
Evaluating alternatives for displaced bunker doors securing radiation protection is ...	26	38	31	6	10
Housing radiotherapy centres in one-floor buildings following strict building codes instead of placing them in multistorey hospital buildings are...	9	24	39	22	17

1: Very important, 2: Important, 3: Neutral, 4: Not important, 5: Not at all important
