# Supplementary material for: Radiotherapy continuity for cancer treatment: Lessons learned from natural disasters
Source: PLoS One. 2025 Sep 3;20(9):e0308056. doi: 10.1371/journal.pone.0308056 (PMC12407550; doi:10.1371/journal.pone.0308056)
Supplement: S4 Text — (PDF) [file pone.0308056.s004.pdf]

Supporting information 4

Structured online questionnaire

The questionnaire, with its questions and response options implemented in the global online survey, is presented below. The questions were mandatory, except for two open-formulated questions at the end of the questionnaire.

Introduction

Dear radiotherapy experts,

My name is Dr. Melanie Reuter-Oppermann and I am an Assistant Professor of the Faculty of Behavioural Management and Social Sciences, University of Twente, in the Netherlands. My research partner, Dr. Ralf Müller-Polyzou, and I investigate the impact of natural hazards on radiotherapy practice. You are the right person for this survey if you work in a radiotherapy centre. Our survey takes only five minutes. We will treat all data anonymously. As a thank you and a sign of dedication to our work, we will personally donate 5 EUR for each completed questionnaire to the German Children's Cancer Foundation. Note: Data will only be sent if the *send button* is used at the end of the survey.

We greatly appreciate your support!

Dr. Melanie Reuter-Oppermann and Dr. Ralf Müller-Polyzou

Basic information

First, we would like to get to know you and your radiotherapy centre.

1. In which country does your radiotherapy centre operate? (drop-down field)

2. In what kind of radiotherapy centre do you work? (single selection)

   a. Public hospital

   b. Private hospital

   c. Private radiotherapy centre

d. Ambulatory radiotherapy centre

e. Others

3. Where is your radiotherapy centre located? (single selection)

a. In a multi-floor building

b. In a single-floor building

c. Other

4. Please indicate your job role. (single selection)

a. Radiation oncologist

b. Medical physicist or dosimetrist

c. Radiation therapist

d. Administration

e. Other

Risk assessment

We are interested in your assessment of risks to business activities in your region.

Note: Business in general, not only radiotherapy.

5. Which natural hazards are most likely to pose the biggest threat in your region, considering a two-year timeframe? (multiple selections possible, except item *None*)

a. Thunderstorm, tornado, hurricane, typhoon

b. Cold and heat waves, fog, hail, drought, dust storms

c. Flood

d. Snow avalanche, glacier hazards

e. Storm surge, sea-level change

f. Earthquake, volcanism

g. Landslide, rock fall, sinkholes, tsunami

h. Wildfire, bushfire

i. None

6. Which natural hazards are most likely to pose the biggest threat in your region, considering a ten-year timeframe? (multiple selections possible, except item *None*)

a. Thunderstorm, tornado, hurricane, typhoon

b. Cold and heat waves, fog, hail, drought, dust storms

c. Flood

d. Snow avalanche, glacier hazards

e. Storm surge, sea-level change

f. Earthquake, volcanism

g. Landslide, rock fall, sinkholes, tsunami

h. Wildfire, bushfire

i. None

7. I believe the following risks are greater in my country than those from natural hazards. (single selection per line)

Cyberattack

a. Strongly Agree

b. Agree

c. Neutral

d. Disagree

e. Strongly Disagree

f. Don't Know

War, terrorism

a. Strongly Agree
b. Agree
c. Neutral
d. Disagree
e. Strongly Disagree
f. Don't Know

Political instability

a. Strongly Agree
b. Agree
c. Neutral
d. Disagree
e. Strongly Disagree
f. Don't Know

Risk prepardness

Please indicate your level of agreement with the following statements regarding the risk to your radiotherapy centre.

8. I know the potential risks of the following hazards for my radiotherapy centre. (single selection per line)

Thunderstorm, tornado, hurricane, typhoon

a. Strongly Agree
b. Agree
c. Neutral
d. Disagree

e. Strongly Disagree

f. Don’t Know

Cold and heat waves, fog, hail, drought, dust stroms

a. Strongly Agree

b. Agree

c. Neutral

d. Disagree

e. Strongly Disagree

f. Don’t Know

Flood

a. Strongly Agree

b. Agree

c. Neutral

d. Disagree

e. Strongly Disagree

f. Don’t Know

Snow avalanche, glacier, hazards

a. Strongly Agree

b. Agree

c. Neutral

d. Disagree

e. Strongly Disagree

f. Don't Know

Storm surge, sea-level change

a. Strongly Agree

b. Agree

c. Neutral

d. Disagree

e. Strongly Disagree

f. Don't Know

Earthquake, volcanism

a. Strongly Agree

b. Agree

c. Neutral

d. Disagree

e. Strongly Disagree

f. Don't Know

Landslide, rock fall, sinkholes, tsunami

a. Strongly Agree

b. Agree

c. Neutral

d. Disagree

e. Strongly Disagree

f. Don't Know

Wildfire, bushfire

a. Strongly Agree

b. Agree

c. Neutral

d. Disagree

e. Strongly Disagree

f. Don't Know

9. I believe that our radiotherapy centre is adequately prepared to handle the risks of ... (single selection per line)

Natural hazards

a. Strongly Agree

b. Agree

c. Neutral

d. Disagree

e. Strongly Disagree

f. Don't Know

Cyberattacks

a. Strongly Agree

b. Agree

c. Neutral

d. Disagree

e. Strongly Disagree

f. Don't Know

War or terrorism

a. Strongly Agree

b. Agree

c. Neutral

d. Disagree

e. Strongly Disagree

f. Don't Know

Political instability

a. Strongly Agree

b. Agree

c. Neutral

d. Disagree

e. Strongly Disagree

f. Don't Know

Risk mitigation

Please rate the following *organisational* measures for natural disaster risk mitigation. Note: According to your personal perception.

10. I believe creating a collaborative network of radiotherapy centres with regional task forces and healthcare coalitions is ... (single selection)

a. Very important

b. Important

c. Neutral

d. Not important

e. Not at all important

11. In my opinion, transferring patients to cooperating radiotherapy centres to continue treatment based on shared health records and considering offering housing and interpretation services is ... (single selection)

a. Very important

b. Important

c. Neutral

d. Not important

e. Not at all important

12. I thnk to organise with the help of radiotherapy associations volunteer staff and aid workers to support or replace own staff and consider special care teams to support patients at home is ... (single selection)

a. Very important

b. Important

c. Neutral

d. Not important

e. Not at all important

13. I believe engaging with vendors, partners, and insurance companies to support emergency offers is ... (single selection)

a. Very important

b. Important

c. Neutral

d. Not important

e. Not at all important

Please rate the following *communication* measures for natural disaster risk mitigation. Note: According to your personal perception.

14. In my opinion, considering alternative communication methods towards patients, such as Social Media and information provision via the Internet or radio, and preparing for psychological support in patient communication is ... (single selection)

a. Very important

b. Important

c. Neutral

d. Not important

e. Not at all important

15. I believe, securing backup communication for leadership and emergency plan execution and preparing for psychological support for caregivers while maintaining an overall positive attitude is ... (single selection)

a. Very important

b. Important

c. Neutral

d. Not important

e. Not at all important

16. In my opinion, securing backup communication methods for communication between caregivers is ... (single selection)

a. Very important

b. Important

c. Neutral

d. Not important

e. Not at all important

Please rate the following *access, protection and therapy* measures for natural disaster risk mitigation. Note: According to your personal perception.

17. I think, offering free transportation for patients to radiotherapy centres, utilising reserved fuel stocks is ... (single selection)

a. Very important

b. Important

c. Neutral

d. Not important

e. Not at all important

18. In my opinion, assuring patients' safety and acute needs and safeguarding inpatients to ensure uninterrupted access to care is ... (single selection)

a. Very important

b. Important

c. Neutral

d. Not important

e. Not at all important

19. I think protecting patients and staff by executing prepared evacuation plans is ... (single selection)

a. Very important

b. Important

c. Neutral

d. Not important

e. Not at all important

20. I think, extending the centre's operation hours to evenings and weekends is ... (single selection)

a. Very important

b. Important

c. Neutral

d. Not important

e. Not at all important

21. In my opinion, compensating additional treatment fractions or using hypofractionation techniques to reduce the number of treatment fractions is ... (single selection)

a. Very important

b. Important

c. Neutral

d. Not important

e. Not at all important

22. I think, pausing scheduled radiotherapy treatments and conduct quality assurances of medical devices before resuming treatments is ... (single selection)

a. Very important

b. Important

c. Neutral

d. Not important

e. Not at all important

Finally, please rate the following *facility and data* measures for natural disaster risk mitigation. Note: According to your personal perception.

23. In my opinion, securing and shutting down sensitive equipment and materials in a controlled manner and protecting against harm, particularly by potential flooding, is ... (single selection)

a. Very important

b. Important

c. Neutral

d. Not important

e. Not at all important

24. In my opinion, housing radiotherapy centres in one-floor buildings following strict building codes instead of placing them in multistorey hospital buildings is ... (single selection)

a. Very important

b. Important

c. Neutral

d. Not important

e. Not at all important

25. I believe, to twin linear accelerators to minimise the need to recalculate treatment plans in case of device loss is ... (single selection)

a. Very important

b. Important

c. Neutral

d. Not important

e. Not at all important

26. In my opinion, securing electricity supply with an emergency generator, not shared with others, with sufficient fuel storage, and protected against direct and indirect disaster impact is ... (single selection)

a. Very important

b. Important

c. Neutral

d. Not important

e. Not at all important

27. I think, offering accommodation for those unable to commute or being evacuated is ... (single selection)

a. Very important

b. Important

c. Neutral

d. Not important

e. Not at all important

28. In my opinion, evaluating alternatives for displaced bunker doors securing radiation protection is ... (single selection)

a. Very important

b. Important

c. Neutral

d. Not important

e. Not at all important

29. I believe, using Electronic Health Records (EHR) with tested recoverable backups in an online repository and data links to cooperating radiotherapy centres is ... (single selection)

a. Very important

b. Important

c. Neutral

d. Not important

e. Not at all important

30. I believe providing the patient with updated records during their treatment course is ... (single selection)

a. Very important

b. Important

c. Neutral

d. Not important

e. Not at all important

Closure

Concluding, did we miss any relevant points? (free text field)

Do you have further comments or suggestions? (free text field)

End of the questionnaire.
