# Supplementary material for: Radiotherapy continuity for cancer treatment: Lessons learned from natural disasters
Source: PLoS One. 2025 Sep 3;20(9):e0308056. doi: 10.1371/journal.pone.0308056 (PMC12407550; doi:10.1371/journal.pone.0308056)
Supplement: S5 Text — (PDF) [file pone.0308056.s005.pdf]

Supporting information 5

Survey data file

The complete dataset from the online survey is available for download under the *Creative Commons Attribution 4.0 International license (CC BY 4.0)* at the following DOI: 10.5281/zenodo.14329747.
